# Cell-intrinsic mechanism involving Siglec-5 associated with divergent outcomes of HIV-1 infection in human and chimpanzee CD4 T cells

**DOI:** 10.1007/s00109-012-0951-7

**Published:** 2012-09-04

**Authors:** Paula C. Soto, Maile Y. Karris, Celsa A. Spina, Douglas D. Richman, Ajit Varki

**Affiliations:** 1 Department of Pathology, University of California, San Diego and Veterans Affairs San Diego Healthcare System, La Jolla, CA USA; 2 Department of Medicine, University of California, San Diego and Veterans Affairs San Diego Healthcare System, La Jolla, CA USA; 3 Department of Pathology and Medicine, University of California, San Diego and Veterans Affairs San Diego Healthcare System, La Jolla, CA USA; 4 Department of Medicine & Cellular and Molecular Medicine, University of California, San Diego, La Jolla, CA USA

**Keywords:** HIV-1 infection, CD4, Siglec-5

## Abstract

Human and chimpanzee CD4+ T cells differ markedly in expression of the inhibitory receptor Siglec-5, which contributes towards differential responses to activating stimuli. While CD4+ T cells from both species are equally susceptible to HIV-1 infection, chimpanzee cells survive better, suggesting a cell-intrinsic difference. We hypothesized that Siglec-5 expression protects T cells from activation-induced and HIV-1-induced cell death. Transduction of human CEM T cells with Siglec-5 decreased cell responses to stimulation. Following HIV-1 infection, a higher percentage of Siglec-5-positive cells survived, suggesting relative resistance to virus-induced cell death. Consistent with this, we observed an increase in percentage of Siglec-5-positive cells surviving in mixed infected cultures. Siglec-5-transduced cells also showed decreased expression of apoptosis-related proteins following infection and reduced susceptibility to Fas-mediated cell death. Similar Siglec-5-dependent differences were seen when comparing infection outcomes in primary CD4+ T cells from humans and chimpanzees. A protective effect of Siglec-5 was further supported by observing greater proportions of circulating CD4+ T cells expressing Siglec-5 in acutely infected HIV-1 patients, compared to controls. Taken together, our results suggest that Siglec-5 expression protects T cells from HIV-1- and apoptosis-induced cell death and contributes to the different outcomes of HIV-1 infection in humans and chimpanzees.

## Introduction

Nonhuman primate models have contributed greatly to our understanding of HIV-1 pathogenesis and have been invaluable for testing the efficacy of experimental vaccines and drug regimens [1, 2]. There are, however, surprising differences in outcomes of HIV-1 infection between humans and captive chimpanzees.

The major circulating clade of HIV-1 arose via cross-species transfer of a chimpanzee SIVcpz and ultimately resulted in rapid progression to AIDS in the great majority of untreated infected humans [3]. Endemic SIVcpz infection in wild chimpanzees can cause a less severe AIDS-like syndrome, with CD4+ T cell depletion, lymphatic tissue destruction, and premature death [4]. However, experimental infections of chimpanzees with HIV-1 only rarely caused AIDS [5], with the great majority of HIV-1-infected chimpanzees still surviving >20 years later. Several explanations have been put forward, including a lack of CD4+ T cell bystander apoptosis, a relative resistance of chimpanzee monocyte macrophages to infection with primary isolates, low HIV-1-specific cytotoxic T lymphocyte responses, low immune activation, and preservation of CD4+ T helper function (reviewed in [6]).

Experimental SIV infection in other nonhuman primate models has led to a better understanding of the pathogenesis of HIV-1 in humans (recently reviewed in [7]). For example, it appears that hyperimmune activation that occurs in patients infected with HIV-1 is driven by factors such as loss of gut-associated CD4+ T cells with loss of GI mucosal integrity and translocation of microbial products, differential expression of pro-inflammatory factors, and differences in upregulation of HIV coreceptors [8]. These systemic factors are doubtless important, but cell-intrinsic factors involving the infected CD4+ T cells themselves also contribute.

One host difference between human and chimpanzee CD4+ T cells is much higher expression of inhibitory Siglecs (sialic acid-binding immunoglobulin-like lectins) in the latter [9]. Siglecs are a subfamily of I-type lectins capable of modulating immune cell activation and death. Most of the CD33-related Siglecs have inhibitory ITIM motifs in their cytosolic tails, which recruit Src homology region 2 domain-containing phosphatases (SHPs) SHP-1 and SHP-2, thus limiting activation pathways stimulated by tyrosine kinases [10].

Siglecs also appear to be involved in apoptotic and nonapoptotic cell death and may regulate immune responses by modulating the life span of cells of the innate immune system [11]. Siglec-10, for example, protects host cells against lethal responses to pathological cell death and helps discriminate danger versus pathogen-associated molecular patterns [12]. In the context of HIV-1 infection, a recent study showed upregulation of Siglec-6 on tissue-like memory B cells of infected individuals [13].

Prior to the availability of antiviral treatments, HIV-1 infection in humans almost universally led to AIDS and death within less than 5–10 years. The marked difference in outcomes of humans versus captive chimpanzees infected with HIV-1 remains unexplained and is likely due to complex and multifaceted interactions between virus and host-specific factors. We have recently shown, using multiple approaches, that chimpanzee T cells are less responsive to stimulation than human T cells and that this difference correlates with Siglec-5 expression [14]. Based on these observations, and on the fact that CD4+ T cells from chimpanzees and humans are equally susceptible to HIV-1 infection in vitro [15, 16], we sought to determine whether a Siglec-mediated cell-intrinsic mechanism contributes to differences in outcomes of HIV-1 infection in CD4+ T cells.

## Materials and methods

### CEM cell culture and nucleofection

The CEM T leukemia cell line (American Type Culture Collection, Manassas, VA) was maintained in RPMI 1640 medium (Gibco-Invitrogen, Carlsbad, CA) supplemented with 1 mM l-glutamine, 50 U/mL penicillin, 50 μg/mL streptomycin, and 10 % (vol/vol) fetal bovine serum (complete medium). For generation of stable clones expressing Siglec-5, 5 × 10^6^ cells were nucleofected using the Human T cell Nucleofector Kit (Amaxa, Gaithersburg, MD) according to the manufacturer's protocol. Cells were then incubated for 72 h and analyzed for Siglec-5 expression. The Siglec-5-positive population was then sorted by flow cytometry (MoFlo, XDP, Beckman Coulter, Brea, CA) into Siglec-5-positive and negative subpopulations.

### Primary CD4+ lymphocyte isolation and stimulation

Chimpanzee blood samples were collected into EDTA-containing tubes at the Yerkes National Primate Center (Atlanta, GA) and shipped on ice overnight to the University of California San Diego, La Jolla, CA. Human blood was collected from healthy volunteer donors, with approval from the University of California San Diego Institutional Review Board. These samples were collected into identical EDTA-containing tubes and stored overnight on ice to ensure similar treatment conditions. Total CD4+ T cells were isolated by negative selection from buffy coats using the RosetteSep cell separation procedure (StemCell Technologies, Vancouver, BC, Canada) followed by density gradient centrifugation with Histopaque 1077 (Sigma-Aldrich, St. Louis, MO). Resulting cell preparations were routinely >95 % positive for CD4+ expression by flow cytometry. The isolated cells were cultured in complete medium. To induce cell stimulation, 2–4 × 10^6^ cells per 2 mL medium were distributed into flat-bottom wells of a six-well, nontissue culture-treated plate that was precoated with anti-CD3 (5–50 ng/mL, clone HIT3a) plus anti-CD28 (0.2 μg/mL) monoclonal antibodies (BD Biosciences, San Jose, CA). The cells were maintained at a concentration of 3–5 × 10^6^ cells per well in complete medium for the remainder of the culture period (13–14 days).

### Cell staining and flow cytometry analysis

The purity of CD4+ lymphocyte preparations and Siglec-5 expression were monitored by direct staining with FITC-conjugated anti-CD4 and/or APC-conjugated anti-Siglec-5 antibodies (BD Biosciences). TRAIL/DR5 expression was monitored using PE-conjugated anti-TRAIL and anti-DR5 antibodies (eBioscience, San Diego, CA). Simple cell surface staining was performed for 30 min at 4 °C per manufacturer's instructions with cell aliquots containing 0.5 × 10^6^ to 1 × 10^6^ cells. For intracellular HIV Gag (p24) analysis, cells were washed in calcium-free and magnesium-free Dulbecco's phosphate-buffered saline (PBS; Mediatech, Herndon, VA), then fixed and permeabilized following the manufacturer's instructions with Cytofix/Cytoperm Buffer (BD Pharmingen, San Diego, CA). After washing with Perm/Wash Buffer (BD Pharmingen), cells were incubated for 30 min with KC57-RD1 antibody (Beckman Coulter, Fullerton, CA). Cells were then washed again with Perm/Wash buffer and stored in 0.5 % formaldehyde at 4 °C until data acquisition on a FACSCalibur (BD Biosciences). For CFSE labeling, 1–2 × 10^6^ PBMCs were labeled with 5 μM CFSE (Invitrogen, Carlsbad, CA), incubated for 15 min at 37 °C, rested on ice for 5 min, and then washed. CFSE content was analyzed by flow cytometry at various time points. Cell viability at the end of culture was monitored using ethidium homodimer-1 staining, following the manufacturer's protocol (LIVE/DEAD Viability/Cytotoxicity kit, Invitrogen). Data were analyzed with FlowJo software.

### Virus infection

Infectious virus stocks of the NL4-3 strain of HIV-1 were prepared by transfecting plasmid DNA (NIH AIDS Research and Reference Reagent Program, Germantown, MD) into the CEM T cells with Lipofectin Transfection Reagent (Invitrogen) and harvesting supernate at the time of peak viral replication and spread. Aliquots of 2–4 × 10^6^ cells were incubated with 0.5 mL of the NL4-3 virus stock for 4–6 h at 37 °C at multiplicities of infection of 0.01 to 0.5 TCID_50_ per cell. After infection, excess virus was removed by extensive washing with PBS plus 2 % fetal calf serum. Cells were stimulated as described above, and virus infection was monitored at different time points during culture. Productive HIV replication was assessed by quantifying the amount of soluble p24 antigen released into culture supernatants by ELISA (Abbott, Abbott Park, IL, and PerkinElmer, Boston, MA).

### Immunoreceptor phosphorylation and cell death-related proteome arrays

The Human Phospho-immunoreceptor Array and the Human Apoptosis Antibody Array (both from R&D Systems, Minneapolis, MN, USA) allow for the simultaneous detection of 59 different phosphorylated immunoreceptors or the relative expression of 35 apoptosis-related proteins. Briefly, cell lysates from CEM cells were diluted with array buffer, added to a four-well multidish and incubated overnight at 2–8 °C. After washing, antibody conjugated to horseradish peroxidase was added, and chemiluminescence was quantified after 2 h by scanning the developed X-ray film on a transmission-mode scanner according to the manufacturer's recommendations.

### Susceptibility to anti-Fas-mediated cell death

1 × 10^5^ CEM cells were plated into each well of a 96-well tissue culture plate and treated with varying concentrations (5–20 μg/mL) of an activating clone of anti-Fas antibody (clone CH11, Millipore, Temecula, CA). The percent killing was calculated by comparing the number of viable cells in untreated wells versus the number of viable cells in treated wells after 24 h in culture.

### HIV-infected patient sample collection and analysis

The San Diego First Choice program was approved by the local Human Research Protections Program and enrolled participants from July 2010 to April 2011. At enrollment, participants underwent a point-of-care rapid antibody test (Oraquick Advance rapid HIV, OraSure Technologies, Inc., Bethlehem, PA). If this test was positive, then confirmation and HIV staging was performed using Western blot (Cambridge Biotech, distributed by Ortho Diagnostics), plasma viral load determination (Cobas Amplicor HIV-1 test, Roche Molecular Systems, Pleasanton, CA), and detuned HIV EIA (Vironostika LS EIA, bioMerieux Inc, Durham, NC, and Vitros LS EIA, Ortho-Clinical Diagnostics, Inc., UK). All study participants had 2 mL whole blood drawn into an EDTA test tube and washed with Wash Buffer (1 L PBS, 10 mL 1 M sodium azide, 20 mL filtered heat-inactivated fetal bovine serum, adjusted to pH 7.2), then centrifuged for 5 min at 300×*g* to allow collection of the buffy coat layer. Washed whole blood (100 μL) was then stained with a fluorescent Ab mixture (FITC/Siglec-5, PE/CD45RO, Per-CP/CD4, PE-Cy7/CD38, APC/CD27, APC-Cy7/CD3, and Pac-Blue/CD8) before evaluation on a FACS Canto II (BD Biosciences, San Jose, CA). Acquired data were compensated using DIVA software, and analysis was performed with FlowJo software (Ashland, OR).

## Results

### CEM cells expressing Siglec-5 survive better in HIV-1-infected cultures

**Fig. 1:**
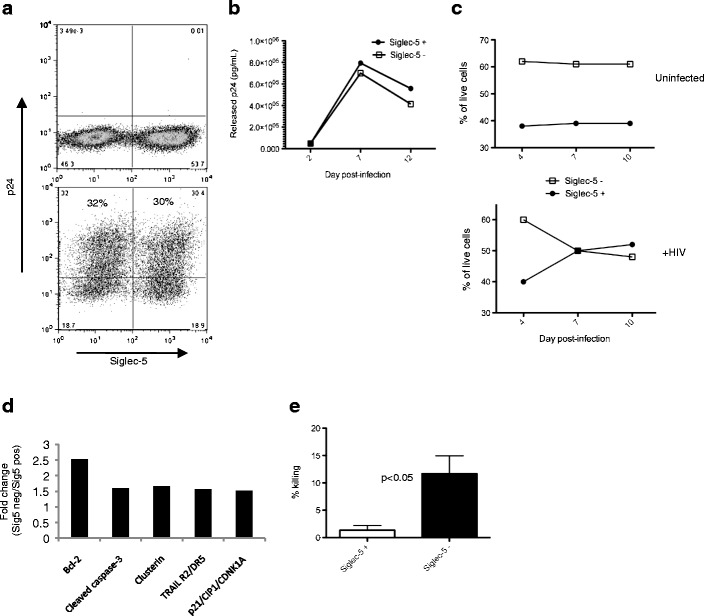
CEM cells expressing Siglec-5 survive better in HIV-1-infected cultures. Mixed population of CEM cells positive or negative for Siglec-5 expression were infected with the NL4-3 clone of HIV. **a** 7 days later, uninfected control (*top*) and infected cells (*bottom*) were stained for intracellular p24 expression and analyzed by flow cytometry. **b** Supernatant from Siglec-5 positive (*closed circles*) and Siglec-5 negative (*open squares*) was collected at multiple time points after HIV-1 infection and analyzed for p24 content by ELISA. **c** Percentages of Siglec-5-positive versus Siglec-5-negative cells in uninfected and infected cultures over time (only live cells as determined by FSC and SSC gating are included in the analysis). **d** Cells were collected on day 7, and relative expression of different apoptosis-related proteins was measured using an antibody array. The graph depicts proteins that showed greater than 1.5-fold difference in expression in Siglec-5-negative cells over Siglec-5-positive cells. **e** CEM cells were treated with an activating anti-Fas antibody for 24 h. Percent killing was determined by comparing the number of viable cells in treated versus untreated wells. *Error bars* represent SEM, *n* = 3

We have previously shown that human and chimpanzee cells differ in expression levels of Siglec-5 and that higher expression correlates with decreased response to activating stimuli [14]. We hypothesized that Siglec-5 would also protect T cells from HIV-1-induced cell death, by dampening responses of activated cells to virus infection. To address this, a mixed population of CEM cells positive and negative for Siglec-5 expression was infected in vitro with the HIV_NL4-3_ clone. Productive infection was evaluated in each Siglec-5 subpopulation by measuring released HIV Gag (p24) at different time points and also by detecting the expression of intracellular p24 7 days after infection. Both showed similar levels of productive infection, with ∼30 % of cells expressing intracellular p24 (Fig. 1a) and similar levels of p24 being released in the culture supernatant (Fig. 1b). The percentage of cells positive for Siglec-5 increased over time in HIV-1-infected cultures, suggesting that Siglec-5-positive cells show enhanced survival following HIV-1 infection (Fig. 1c). To further support the hypothesis that Siglec-5 expression enhanced cell survival and to identify a potential mechanism, we measured relative expression of 35 apoptosis-related proteins in Siglec-5-positive and negative CEM subsets. Siglec-5-negative cells, some of which were infected with HIV-1, showed increased expression of several apoptosis-related proteins, including the active form of caspase-3, and TRAILR2/DR5 (Fig. 1d). Siglec-5-positive cells were also more resistant to Fas-mediated cell death, induced by antibody cross-linking and activation of the membrane Fas (Fig. 1e). These observations support the hypothesis that Siglec-5 expression, either directly or indirectly, by affecting the expression of other cofactors, enhances T cell survival during HIV infection.

### Siglec-5 expression in CEM T cells alters the pattern of immunoreceptor phosphorylation and decreases TNF production in response to stimulation

**Fig. 2:**
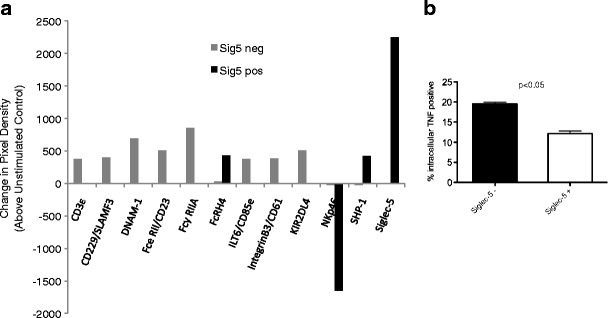
Divergent pattern of immunoreceptor phosphorylation and decreased TNF production in response to stimulation in CEM cells expressing Siglec-5. CEM cells were nucleofected with a Siglec-5 expression plasmid and sorted into homogeneous populations of Siglec-5-positive and Siglec-5-negative cells. **a** Cells were activated with PHA for 4 h and the cell lysate collected. Lysate from stimulated and unstimulated cells was added to phospho-immunoreceptor antibody array, and immunoblot image analyzed. The graph shows a change in pixel density of stimulated cells above unstimulated cell control. **b** Mixed population of CEM cells positive or negative for Siglec-5 expression were stimulated with PMA and anti-CD3. Intracellular TNF production was analyzed 4 h later by flow cytometry. *Error bars* represent SEM, *n* = 4

In order to identify potential pathways involved, CEM T cells were transfected with a Siglec-5 expression plasmid and activated with PHA. The relative phosphorylation of >50 immunoreceptors in cells with or without activation was then analyzed using an immunoblot array. The CEM subset expressing Siglec-5 had fewer immunoreceptors showing tyrosine phosphorylation (Fig. 2a), suggesting a dampened response to PHA stimulation. Of note, Siglec-5 itself was phosphorylated upon activation. Consistent with an inhibitory role, expression of Siglec-5 in CEM cells also resulted in lower percentages of cells expressing TNF following anti-CD3/PMA stimulation (Fig. 2b).

### Chimpanzee CD4+ T cells with Siglec-5 expression survive better than human CD4+ T cells following HIV-1 infection

**Fig. 3:**
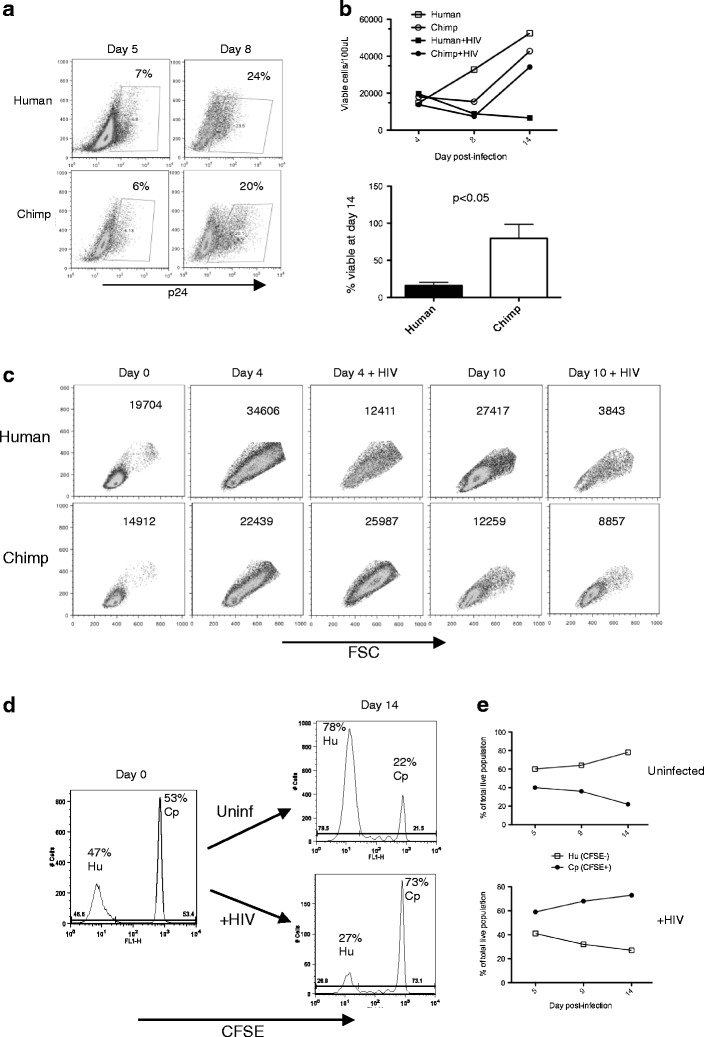
Chimpanzee CD4+ T cells expressing Siglec-5 survive better than human CD4+ T cells in HIV-1-infected cultures. Human and chimpanzee CD4+ T cells were isolated from whole blood, infected with HIV-1, and stimulated with anti-CD3/CD28. **a** Intracellular p24 expression was monitored on days 5 and 8 postinfection by flow cytometry. **b** *Top panel*: representative absolute numbers of viable cells in human- and chimpanzee-infected or uninfected cultures over time. *Bottom panel*: The percentage of live cells at day 14 of culture was determined by flow cytometry (*n* = 5, *error bars* represent SEM). **c** Representative panels of human and chimpanzee cultures with and without HIV infection. *Numbers* on the *top right corner* represent absolute number of viable cells (as determined by forward and side scatter gating) collected for a specified amount of time. Data representative of three different comparisons. **d** Chimpanzee CD4+ T cells were labeled with CFSE and then mixed at a ratio of 1:1 with human CD4+ T cells, infected with HIV or not as a control, and stimulated with anti-CD3/CD28. Percentage of cells in each population (CFSE+ and CFSE−) was monitored for 14 days. Histograms depict the percentage of live cells that are CFSE positive and CFSE negative at day 0 and at the end of culture (day 14). **e** Graphs depict the percentage of each population (CFSE+ and CFSE−) over time in uninfected cultures (*top*) and cultures infected with HIV-1 (*bottom*). Data shown are representative of three experiments

We next asked if similar results would be observed in primary T cells that naturally express Siglec-5. CD4+ T cells were isolated from human (more than 99 % Siglec-5 negative) and chimpanzee (more than 99 % Siglec-5 positive) whole blood and infected with HIV-1, and both human and chimpanzee cells were stimulated with a high dose of anti-CD3/CD28 antibodies. Productive viral infection and cell viability were monitored on multiple days postinfection. The percentage of CD4+ T cells productively infected did not differ significantly between human and chimpanzee samples (Fig. 3a). There was, however, a significant difference in the number of surviving cells at the end of culture (14 days postinfection), with chimpanzee cells having a significantly better rate of survival (Fig. 3b and c). To determine if the increased survival in chimpanzee cell cultures was due to cell-intrinsic or cell-extrinsic mechanisms, chimpanzee CD4+ T cells were labeled with CFSE and then mixed at a ratio of 1:1 with unlabeled human CD4+ T cells. A portion of the mixed culture was infected with HIV, both infected and uninfected cultures were stimulated with anti-CD3/CD28 antibodies, and the relative percentage of cells in each population (CFSE+ and CFSE−) was monitored for 14 days. As shown in Fig. 3d and e, chimpanzee cells survived better following HIV-1 infection, even within the same culture as human cells. In striking contrast, there was an increase in percentage of human cells in the absence of HIV infection, due to their increased proliferation, as anticipated from our previous work. These results point to a cell-intrinsic mechanism being responsible for the increased survival of chimpanzee cells following HIV-1 infection.

### Increased cell death of HIV-infected human CD4+ T cells is associated with increased expression of apoptosis-related proteins

**Fig. 4:**
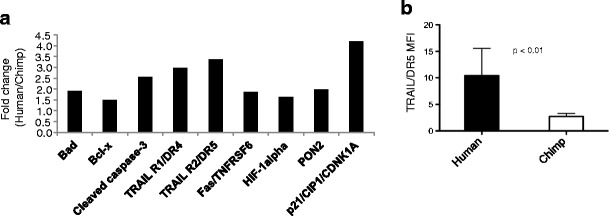
Increased cell death of human CD4+ T cell is associated with increased expression of apoptosis-related proteins. HIV-1-infected human and chimpanzee CD4+ T cells were collected 8 days after infection and analyzed for expression of apoptosis-related proteins. **a** Relative expression of different apoptosis-related proteins was measured using an antibody array. The graph depicts proteins that showed greater than 1.5-fold difference in increased expression in HIV-1-infected human over chimpanzee CD4+ T cells. One comparison with 2 × 10^6^ cells from each species was performed. **b** Surface expression of TRAIL and DR5 were analyzed at day 8 postinfection in HIV-1-infected human and chimpanzee CD4+ T cells. *Error bars* represent SEM, *n* = 4

Next, we examined potential apoptosis-related signaling differences between HIV-1-infected human and chimpanzee CD4+ T cells, by analyzing the expression profiles of 35 apoptosis-related proteins using a proteome array. In agreement with data obtained with CEM cells expressing Siglec-5, we observed lower levels of expression of the active form of caspase-3, Fas, and TRAIL receptors 1 and 2 in chimpanzee cells that naturally express Siglec-5 (Fig. 4a), when compared to expression levels in human cells. Chimpanzee cells also demonstrated significantly lower surface level expression of DR5 and TRAIL 10 days after infection (Fig. 4b), in agreement with previous reports showing increased susceptibility to apoptosis in human cells when compared to chimpanzee cells [16, 17]. Attempts at siRNA-mediated knockdown of Siglec-5 did not work in primary chimpanzee T cells, due to upregulation of Siglec-5 mRNA following cell activation, and the toxicity of siRNAs in primary cells in long-term studies (data not shown).

### Enrichment of CD4+ T cells expressing Siglecs in human CD4+ T cell cultures infected with HIV-1 and in HIV-1-infected patient samples

**Fig. 5:**
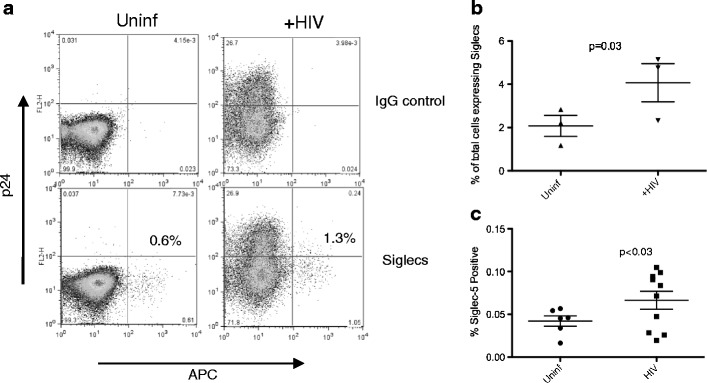
Enrichment of CD4+ T cells expressing Siglecs in human CD4+ T cell cultures infected with HIV-1 and in HIV-1-infected patient samples. **a** Purified primary human CD4+ T cells were infected with HIV-1 or left uninfected as a control, and stimulated with anti-CD3/CD28. After 8 days, the percentage of CD4+ T cells expressing intracellular p24 and both inhibitory Siglecs 5 and 9 was analyzed by flow cytometry and compared to the percentage of Siglec-expressing cells in uninfected cultures. **b** The graph depicts summary data of HIV-1 infection of CD4+ T cells from three different individuals as described above. The data shown represent the percentage of total cells expressing Siglecs 8 days postinfection. **c** The percentage of CD3+CD4+ cells also positive for Siglec-5 expression in PBMCs from patients with recent HIV infection (*n* = 10) was analyzed by flow cytometry and compared to samples from uninfected healthy donors (*n* = 6). *Error bars* represent SEM

To directly assess whether Siglec expression in human CD4+ T cells would improve cell survival, we took advantage of the fact that a minor subset of CD4+ T cells (usually less than 1 %) in some humans do express members of the CD33-related family of receptors. We hypothesized that this minor subpopulation would be enriched following HIV-1 infection. In order to increase the detection of such a minor population, cells were stained for expression of two members of the Siglec family: Siglec-5 and Siglec-9, both of which are inhibitory. Purified human CD4 T cells were infected with HIV-1, stimulated with anti-CD3/CD28, and cultured for 8 days, and the percentage of CD4+ T cells expressing intracellular p24 and/or Siglecs 5 and 9 was analyzed by flow cytometry and compared to the cells expressing Siglecs in uninfected cultures. Cultures from three different individuals showed an increase in the small percentage of Siglec-expressing cells following HIV-1 infection (Fig. 5a and b). The data suggest that Siglec-expressing cells are better able to survive in HIV-1-infected cultures, resulting in enrichment of these cells, as the majority of cells dying are non-Siglec-expressing cells. We do not believe that Siglec-5-positive cells are expanding with HIV infection, since expression of Siglec-5 in human cells is strongly repressed, as suggested by our prior studies, which have shown that Siglec-5 expression is not induced upon cell activation nor upon treatment with certain epigenetic modifying drugs (data not shown). It is still possible, however, that in the three individuals used in our study, Siglec expression may not be as strongly repressed as in other individuals.

To determine if enrichment of Siglec-5-positive cells also occurs in vivo, the percentage of CD4+ cells with Siglec-5 expression was analyzed by flow cytometry in peripheral blood samples from patients with recent HIV infection (Table 1). Compared to HIV-negative controls, such patients demonstrated a significant increase in percentage of CD4+ T cells that expressed Siglec-5 (Fig. 5c).

**Table 1:** Estimated date of infection, CD4 counts, and viral load for HIV-1-positive individuals

Patient number	Days from EDI^a^	CD4 counts (cells/mL)	HIV plasma viral load (log10 copies/mL)
1	19	401	5.5
2	91	870	3.0
3	25	362	5.7
4	76	521	3.8
5	20	425	4.5
6	106	471	5.8
7	29	897	4.9
8	102	862	4.9
9	35	609	3.2
10	75	698	6.2

^a^Estimated date of infection. All patients were antiretroviral naive

## Discussion

Since the discovery of HIV-1 as the causative agent of AIDS, the use of chimpanzees as disease models has been insightful, challenging, and controversial. Based on the fact that chimpanzees were equally susceptible to infection by HIV, there was great hope that they would be useful as an animal model for disease pathogenesis [18, 19]. However, despite similar infection susceptibility and virus production, the outcome of HIV-1 infection was quite different in these animals [5, 15, 17] with the great majority surviving without AIDS for >20 years and many still alive today. The exception that actually proves the rule was an unusual strain that evolved to cause AIDS in a multiply infected chimp [20] and proved highly pathogenic when transferred to other chimps [21]. Over the years, the literature has accumulated conflicting reports regarding cytopathic effects of HIV in chimpanzee CD4+ T cells, virus replication capacity, and CD8 T cell-killing ability [16, 22–26]. Our study addresses cell-intrinsic differences between human and chimpanzee CD4+ T cells that can clarify differences in their ability to succumb to HIV-1 infection.

Here, we show that a human T cell line expressing Siglec-5 shows both a reduced response to activating stimuli and an intrinsic advantage in cell survival following HIV-1 infection. The decreased response to activating stimuli was associated with higher tyrosine phosphorylation of activating receptors, such as CD3 epsilon and NKp46, in Siglec-5-negative CEM cells. The increased survival of Siglec-5-positive cells in HIV-1-infected cultures was associated with decreased expression of apoptosis-related proteins, like the active form of caspase-3, and TRAILR2/DR5. How does Siglec-5 expression confer an intrinsic advantage in terms of cell survival following HIV-1 infection in vitro? Siglec-5 has been previously shown to mediate SHP-1- and/or SHP-2-dependent signaling even in the absence of tyrosine phosphorylation [27] and may affect T cell survival via this mechanism. More recently, the absence of SHP-1 has been shown to increase Fas-induced cell death [28]. T cells with Siglec-5 expression demonstrated a similar functional phenotype, consistent with SHP-1 recruitment and inhibition of apoptosis. We cannot of course rule out the possibility that Siglec expression is indirectly correlated with protection via some other unknown mechanism. Further work is needed to clarify this relationship.

When compared to HIV-negative controls, recently infected HIV-1-positive individuals had significant enrichment of Siglec-5-positive CD4+ T cells. Interestingly, Siglec-6 is upregulated on tissue-like memory B cells of HIV-viremic individuals [13]. The authors of that study suggest that Siglec expression is a marker of a dysfunctional, anergic phenotype in the setting of immune system exhaustion. However, another possible interpretation is that cells expressing Siglecs preferentially survive the heightened immune activation and activation-induced cell death caused by HIV-1 infection. The differences in functionality of Siglec-positive cells could also reflect their maturational state. In our HIV-infected cohort, Siglec-5 expression was mainly detected on naïve CD4+ T cells (data not shown), which could appear dysfunctional if compared to responses of mature memory and effector T cells. Thus, instead of representing an exhausted phenotype, Siglec expression in this setting may be a marker of differentiation correlating with a less mature phenotype. Further work is needed to understand the mechanisms regulating Siglec-5 expression and its relation to T cell differentiation.

Another recent study [29] concluded that decreased expression of Siglec-7 on natural killer cells represents an early marker of dysfunctional NK-cell subsets, associated with high levels of HIV-1 viremia. These authors suggested that high frequencies of Siglec-7-negative NK cells might reflect the immune and clinical status of HIV-1 infection and can also track the effectiveness of therapy. Interestingly, they also observed maintenance of Siglec-7-positive NK cells in long-term nonprogressors. In this uncommon setting, Siglec expression may also contribute to control of immune activation and delayed disease progression.

The use of lentivirus infection of chimpanzees as a model for understanding HIV pathogenesis has been further complicated by a long-term natural history study of infected wild chimpanzees which revealed that, contrary to what was previously thought, SIVcpz can be pathogenic [4] and may be associated with chimpanzee population decline [30]. Interestingly, the latter study concludes that population extinction is not an inevitable consequence of SIVcpz infection despite negative effects of the virus on population size. Taken together, the data indicate SIVcpz infection in this population is less pathogenic than untreated HIV-1 infection in humans, which was almost always fatal prior to the availability of antiviral treatments.

These recently observed differences in outcome of SIVcpz infection in its natural host may appear contradictory at first, as with in vitro comparisons between human and chimpanzee T cells, but could be explained by a combination of factors. Siglec expression is one of these factors that can tilt the balance in favor of the host but obviously does not offer complete protection. Also, the results obtained from comparisons of human and chimp CD4+ T cells in terms of activation thresholds, susceptibility to infection, and susceptibility to cytopathic effects can be rather different depending on the experimental conditions, i.e., how the cells are activated, for how long, and what type of virus strain is used [31]. Clearly, the factors that contribute to different HIV/SIV infection outcomes in different nonhuman primate species are many, and these differences may be greater than previously thought.

Here, we report on a novel cell-intrinsic factor that may help protect T cells from activation and HIV-induced cell death and supports the role of excessive immune activation as one of the main contributors to CD4 T cell loss. Understanding the pathways involved in dampening immune activation and that result in protection may lead to novel therapeutic strategies.

## References

[1] Staprans SI, Feinberg MB (2004). The roles of nonhuman primates in the preclinical evaluation of candidate AIDS vaccines. Expert Rev Vaccines.

[2] Rompay KK (2011). The use of nonhuman primate models of HIV infection for the evaluation of antiviral strategies. AIDS Res Hum Retroviruses.

[3] Sharp PM, Hahn BH (2010). The evolution of HIV-1 and the origin of AIDS. Philos Trans R Soc Lond B Biol Sci.

[4] Keele BF, Jones JH, Terio KA, Estes JD, Rudicell RS, Wilson ML, Li Y, Learn GH, Beasley TM, Schumacher-Stankey J (2009). Increased mortality and AIDS-like immunopathology in wild chimpanzees infected with SIVcpz. Nature.

[5] Johnson BK, Stone GA, Godec MS, Asher DM, Gajdusek DC, Gibbs CJJ (1993). Long-term observations of human immunodeficiency virus-infected chimpanzees. AIDS Res Hum Retroviruses.

[6] Juompan LY, Hutchinson K, Montefiori DC, Nidtha S, Villinger F, Novembre FJ (2008). Analysis of the immune responses in chimpanzees infected with HIV type 1 isolates. AIDS Res Hum Retroviruses.

[7] Brenchley JM, Paiardini M (2011). Immunodeficiency lentiviral infections in natural and non-natural hosts. Blood.

[8] Brenchley JM, Silvestri G, Douek DC (2010). Nonprogressive and progressive primate immunodeficiency lentivirus infections. Immunity.

[9] Nguyen DH, Hurtado-Ziola N, Gagneux P, Varki A (2006). Loss of Siglec expression on T lymphocytes during human evolution. Proc Natl Acad Sci U S A.

[10] Crocker PR, Paulson JC, Varki A (2007). Siglecs and their roles in the immune system. Nat Rev Immunol.

[11] von Gunten S, Simon HU (2006). Sialic acid binding immunoglobulin-like lectins may regulate innate immune responses by modulating the life span of granulocytes. FASEB J.

[12] Chen GY, Tang J, Zheng P, Liu Y (2009). CD24 and Siglec-10 selectively repress tissue damage-induced immune responses. Science.

[13] Kardava L, Moir S, Wang W, Ho J, Buckner CM, Posada JG, O'Shea MA, Roby G, Chen J, Sohn HW (2011). Attenuation of HIV-associated human B cell exhaustion by siRNA downregulation of inhibitory receptors. J Clin Invest.

[14] Soto PC, Stein LL, Hurtado-Ziola N, Hedrick SM, Varki A (2010). Relative over-reactivity of human versus chimpanzee lymphocytes: implications for the human diseases associated with immune activation. J Immunol.

[15] Heeney J, Bogers W, Buijs L, Dubbes R, ten Haaft P, Koornstra W, Niphuis H, Nara P, Teeuwsen V (1996). Immune strategies utilized by lentivirus infected chimpanzees to resist progression to AIDS. Immunol Lett.

[16] Kim N, Dabrowska A, Jenner RG, Aldovini A (2007). Human and simian immunodeficiency virus-mediated upregulation of the apoptotic factor TRAIL occurs in antigen-presenting cells from AIDS-susceptible but not from AIDS-resistant species. J Virol.

[17] Gougeon ML, Lecoeur H, Boudet F, Ledru E, Marzabal S, Boullier S, Roue R, Nagata S, Heeney J (1997). Lack of chronic immune activation in HIV-infected chimpanzees correlates with the resistance of T cells to Fas/Apo-1 (CD95)-induced apoptosis and preservation of a T helper 1 phenotype. J Immunol.

[18] Alter HJ, Eichberg JW, Masur H, Saxinger WC, Gallo R, Macher AM, Lane HC, Fauci AS (1984). Transmission of HTLV-III infection from human plasma to chimpanzees: an animal model for AIDS. Science.

[19] Fultz PN, McClure HM, Swenson RB, McGrath CR, Brodie A, Getchell JP, Jensen FC, Anderson DC, Broderson JR, Francis DP (1986). Persistent infection of chimpanzees with human T-lymphotropic virus type III/lymphadenopathy-associated virus: a potential model for acquired immunodeficiency syndrome. J Virol.

[20] Novembre FJ, Saucier M, Anderson DC, Klumpp SA, O'Neil SP, Brown CRI, Hart CE, Guenthner PC, Swenson RB, McClure HM (1997). Development of AIDS in a chimpanzee infected with human immunodeficiency virus type 1. J Virol.

[21] O'Neil SP, Novembre FJ, Hill AB, Suwyn C, Hart CE, Evans-Strickfaden T, Anderson DC, deRosayro J, Herndon JG, Saucier M (2000). Progressive infection in a subset of HIV-1-positive chimpanzees. J Infect Dis.

[22] Ondoa P, Davis D, Kestens L, Vereecken C, Garcia RS, Fransen K, Heeney J, van der Groen G (2002). In vitro susceptibility to infection with SIVcpz and HIV-1 is lower in chimpanzee than in human peripheral blood mononuclear cells. J Med Virol.

[23] Pischinger K, Zimmermann K, Eibl MM, Mannhalter JW (1998). Comparison of early events during infection of human and chimpanzee peripheral blood mononuclear cells with HIV-1. Arch Virol.

[24] Saksela K, Muchmore E, Girard M, Fultz P, Baltimore D (1993). High viral load in lymph nodes and latent human immunodeficiency virus (HIV) in peripheral blood cells of HIV-1-infected chimpanzees. J Virol.

[25] Wain LV, Bailes E, Bibollet-Ruche F, Decker JM, Keele BF, Van Heuverswyn F, Li Y, Takehisa J, Ngole EM, Shaw GM (2007). Adaptation of HIV-1 to its human host. Mol Biol Evol.

[26] Zarling JM, Ledbetter JA, Sias J, Fultz P, Eichberg J, Gjerset G, Moran PA (1990). HIV-infected humans, but not chimpanzees, have circulating cytotoxic T lymphocytes that lyse uninfected CD4+ cells. J Immunol.

[27] Avril T, Freeman SD, Attrill H, Clarke RG, Crocker PR (2005). Siglec-5 (CD170) can mediate inhibitory signalling in the absence of immunoreceptor tyrosine-based inhibitory motif phosphorylation. J Biol Chem.

[28] Koncz G, Kerekes K, Chakrabandhu K, Hueber AO (2008). Regulating Vav1 phosphorylation by the SHP-1 tyrosine phosphatase is a fine-tuning mechanism for the negative regulation of DISC formation and Fas-mediated cell death signaling. Cell Death Differ.

[29] Brunetta E, Fogli M, Varchetta S, Bozzo L, Hudspeth KL, Marcenaro E, Moretta A, Mavilio D (2009). The decreased expression of Siglec-7 represents an early marker of dysfunctional natural killer-cell subsets associated with high levels of HIV-1 viremia. Blood.

[30] Rudicell RS, Holland Jones J, Wroblewski EE, Learn GH, Li Y, Robertson JD, Greengrass E, Grossmann F, Kamenya S, Pintea L (2010). Impact of simian immunodeficiency virus infection on chimpanzee population dynamics. PLoS Pathog.

[31] Decker JM, Zammit KP, Easlick JL, Santiago ML, Bonenberger D, Hahn BH, Kutsch O, Bibollet-Ruche F (2009). Effective activation alleviates the replication block of CCR5-tropic HIV-1 in chimpanzee CD4+ lymphocytes. Virology.

